# Frequent Extreme Cold Exposure and Brown Fat and Cold-Induced Thermogenesis: A Study in a Monozygotic Twin

**DOI:** 10.1371/journal.pone.0101653

**Published:** 2014-07-11

**Authors:** Maarten J. Vosselman, Guy H. E. J. Vijgen, Boris R. M. Kingma, Boudewijn Brans, Wouter D. van Marken Lichtenbelt

**Affiliations:** 1 Department of Human Biology, School for Nutrition, Toxicology and Metabolism – NUTRIM, Maastricht, the Netherlands; 2 Department of Nuclear Medicine, Maastricht University Medical Center+, Maastricht, the Netherlands; 3 Department of Surgery (G.V.), Erasmus Medical Center, Rotterdam, the Netherlands; St. Joseph's Hospital and Medical Center, United States of America

## Abstract

**Introduction:**

Mild cold acclimation is known to increase brown adipose tissue (BAT) activity and cold-induced thermogenesis (CIT) in humans. We here tested the effect of a lifestyle with frequent exposure to extreme cold on BAT and CIT in a Dutch man known as ‘the Iceman’, who has multiple world records in withstanding extreme cold challenges. Furthermore, his monozygotic twin brother who has a ‘normal’ sedentary lifestyle without extreme cold exposures was measured.

**Methods:**

The Iceman (subject A) and his brother (subject B) were studied during mild cold (13°C) and thermoneutral conditions (31°C). Measurements included BAT activity and respiratory muscle activity by [^18^F]FDG-PET/CT imaging and energy expenditure through indirect calorimetry. In addition, body temperatures, cardiovascular parameters, skin perfusion, and thermal sensation and comfort were measured. Finally, we determined polymorphisms for uncoupling protein-1 and β3-adrenergic receptor.

**Results:**

Subjects had comparable BAT activity (A: 1144 SUV_total_ and B: 1325 SUV_total_), within the range previously observed in young adult men. They were genotyped with the polymorphism for uncoupling protein-1 (G/G). CIT was relatively high (A: 40.1% and B: 41.9%), but unlike during our previous cold exposure tests in young adult men, here both subjects practiced a g-Tummo like breathing technique, which involves vigorous respiratory muscle activity. This was confirmed by high [^18^F]FDG-uptake in respiratory muscle.

**Conclusion:**

No significant differences were found between the two subjects, indicating that a lifestyle with frequent exposures to extreme cold does not seem to affect BAT activity and CIT. In both subjects, BAT was not higher compared to earlier observations, whereas CIT was very high, suggesting that g-Tummo like breathing during cold exposure may cause additional heat production by vigorous isometric respiratory muscle contraction. The results must be interpreted with caution given the low subject number and the fact that both participants practised the g-Tummo like breathing technique.

## Introduction

During cold exposure the human body may increase heat production by shivering and non-shivering thermogenesis (NST), and minimize heat loss by vasoconstriction [1]. A major tissue responsible for NST is brown adipose tissue (BAT). BAT produces heat via uncoupling protein-1 (UCP-1), which uncouples the respiratory chain from ATP production thereby releasing energy as heat. A lifestyle that includes frequent cold exposure might result in acclimatization and, consequently, a better-equipped thermoregulatory machinery to fight the cold. It is now well established that BAT is still present and functional in human adults during cold exposure [2]–[4]. Furthermore, mild cold acclimatization in humans has shown to increase NST [5], [6] and BAT activity [6]. However, the effects of a lifestyle with frequent exposures to extreme cold conditions on these parameters are unknown.

It has been shown that a great variability in NST and BAT exists within the same population groups, which may be attributed to differences in lifestyle effects [7]. However, a genetic component may influence the capacity for NST as well. For instance, it has been suggested that polymorphisms in the uncoupling protein-1 gene and β3-adrenergic receptor influence resting energy expenditure [8] and accelerate age-related decrease in BAT activity in elderly [9]. It is thus likely that both nature (i.e. genetic make up) and nurture (lifestyle) influence the existence and heat generating potential of BAT. Next to BAT, also other tissues, such as skeletal muscle, might contribute to NST [10].

In this perspective, we report on the thermoregulatory responses of a monozygotic twin. One of the twins, the so-called “Iceman”, has been exposed to frequent periods of extreme cold for several decades. He is world-record holder in withstanding extreme cold exposure under several disciplines, such as the fastest half-marathon on snow and ice while barefoot, and the longest duration while fully immersed in crushed ice (1 hour and 50 minutes). He claims to achieve these records through a special meditation and breathing technique, which is based on g-Tummo meditation, and that he is capable to regulate his own autonomic nervous system. A recent case study demonstrated that he was capable to control the autonomic stress response during endotoxemia by using this technique [11]. His monozygotic twin brother does not experience extreme cold exposure due to a different occupation and a sedentary lifestyle, although he is aware of the techniques used by his brother. We here tested the hypothesis that the iceman has a higher cold-induced heat production and cold-induced BAT activity compared to his identical twin brother. This case study provides a unique comparison between a monozygotic twin pair, one being extremely cold-acclimated the other being not cold-acclimated at all.

## Methods

### Subjects

The Medical Ethical Committee of the Maastricht University Medical Centre+ approved the protocols and both the Iceman (subject A) and his twin brother (subject B) gave written informed consent. All procedures were conducted according to the principles of the Declaration of Helsinki. The monozygotic twin brothers are both male and 52 years of age. They were screened for medical history, and were absent of any factors related to the metabolic syndrome, and had no thyroid gland dysfunction. They were not using any type of medication, which could have affected the results. Subject A is well known for his capability to withstand extreme cold challenges. He is a self-employed lifestyle educator, focusing on the capacity of the human mind to control its body by means of a special meditation and breathing technique (based on g-Tummo) [12]. He has been exposing himself to daily cold water swimming/showers and regularly visits Scandinavian and arctic regions in order to practice and train extreme cold exposure in shorts only. Subject B is employed as an international truck driver, being on the road for multiple days in a row and experiencing longer periods of inactivity. He does not practice and train extreme cold exposure. However, he is familiar with the special meditation and breathing technique used by his brother.

### Study design and measurements

**Figure 1:**
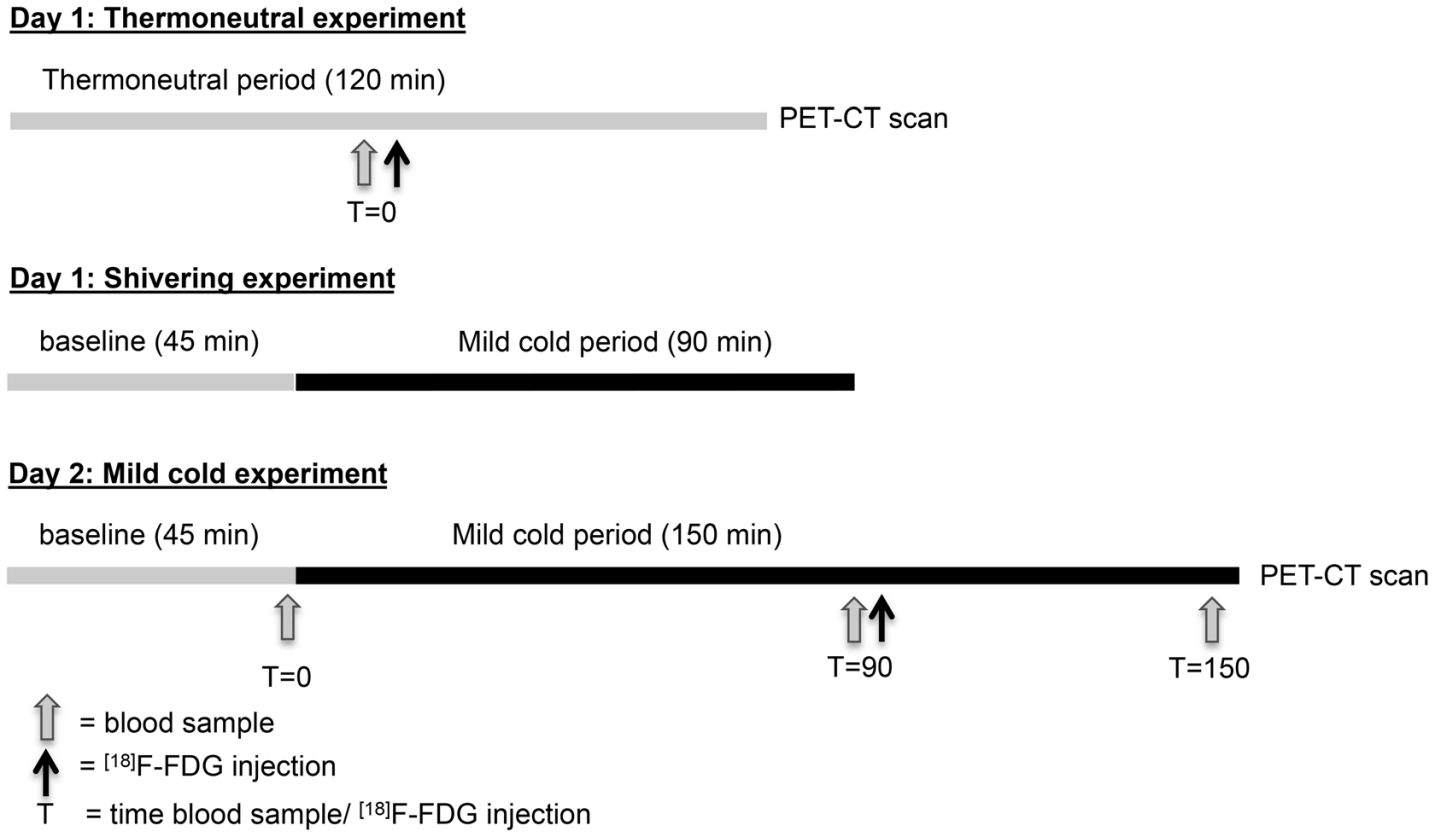
Study protocol. The thermoneutral experiment started in the morning on day one. After one hour a blood sample was taken and subsequently the [^18^F]FDG tracer was injected followed by the PET-CT scan one hour later. In the afternoon of day one, the shivering experiment was conducted, which started with a baseline period of 45 minutes during 31°C followed by 90 minutes of mild cold exposure (31°C) to determine the ambient temperature at which shivering occurred. The mild cold experiment on day two consisted of 45 minutes baseline (31°C) followed by 150 minutes of cold exposure. Blood samples were taken at the end of the baseline period and 90 and 150 minutes after the onset of cold exposure. The [^18^F]FDG tracer was injected 90 minutes after the onset of cold exposure, followed by the PET-CT scan one hour later.

Subjects were measured on two separate occasions during two consecutive days in the winter season with comparable outdoor temperatures. The study protocols in both subjects were identical. In the morning of day one, BAT activity was measured during thermoneutral conditions (i.e. two hours at air temperature 31°C), which served as a control-measurement (**Figure 1**). In the afternoon of day one, a “shivering test” was performed with the purpose to determine the air temperature associated with the onset of shivering. This information was then used for the mild cold experiment on day two to determine the ambient temperature to obtain maximal cold-induced thermogenesis (CIT) (i.e. at the lowest temperature without shivering). The mild cold experiment on day two started with a 45-minute baseline during thermoneutral conditions (∼31°C), followed by two and half hours of mild cold exposure (12–13°C). The [^18^F]fluorodeoxyglucose (FDG) tracer was injected intravenously after 90 minutes in the cold and 60 minutes later the PET-CT scan was performed. The measurements were performed in a specially equipped air-permeable tent (Colorado altitude training, USA), in which ambient temperature was tightly controlled. Subjects were measured in semi-supine position on an air-permeable stretcher (Model Campart Rome-XL) in order to lie comfortably. The subjects wore shorts only (Clo 0.06)[13]. During the experiments we measured energy expenditure via indirect calorimetry (Ventilated Hood, Omnical 2, Maastricht, the Netherlands), heart rate via a monitor on the chest (Polar T31, USA), and blood pressure via a pressure cuff on the left upper arm (Cresta, Taiwan). Mean arterial blood pressure (MAP) was calculated as *MAP  =  1/3 Systolic pressure + 2/3 Diastolic pressure*. Skin temperatures were determined via wireless temperature sensors placed at the 14 sites prescribed by ISO-standard 9886:2004 (Ergonomics – Evaluation of thermal strain by physiological measurements, International Standards Organization, Geneva, Switzerland) (iButtons Maxim integrated, USA), and core temperature via a telemetric pill (CorTemp HT150002, USA), which was ingested one hour before the onset of the experiment. Vasoconstriction was measured by determining the change in skin perfusion via Laser Doppler at the ventral side of the hand at the base of the thumb, at the ventral side of the hallux (Perimed PF4000, Sweden), at the ventral side of the forearm halfway between the elbow and the wrist, and at the abdomen halfway between the umbilicus and the left lateral side of the body (Perimed PF5000, Sweden). Venous blood samples were taken during baseline and in the cold for analysis of hormones and metabolites, and DNA analysis via PCR to identify polymorphisms for uncoupling protein-1 (-3286 A/G polymorphism) [14] and the β3-adrenergic receptor (Trp64Arg) [15].

During the mild cold experiment, both subjects completed visual analogue scales (VAS-scales) [16], on sensation, thermal comfort and shivering. Furthermore, BAT activity was assessed by [^18^F]fluorodeoxyglucose-positron emission-tomography-computed tomography ([^18^F]FDG-PET-CT) (Gemini TF PET-CT, Philips, The Netherlands). The protocol and analysis were comparable to our previous studies using static imaging [17]. A low-dose CT scan (120 kV, 30 mAs) preceded the PET scan, and was used for attenuation and scatter correction of the PET scan. The PET scan was used to determine [^18^F]FDG-uptake. Temperature was regulated with a heater and air-conditioning. Body composition was determined by means of dual x-ray absorptiometry (DXA, Hologic, type Discovery A, USA), and in the afternoon, a biopsy was taken from the m. vastus lateralis for mitochondrial respirometry measurements in permeabilized muscle fibers by means of the Oxygraph-2K (Oroboros, Austria). A part of the biopsy was placed in a preservation medium for the respiration measurements (for substrate details see [6]), and a portion of the muscle tissue was immediately frozen in melting isopentane and stored at −80°C for determination of mitochondrial DNA (mtDNA) copy number (ratio ND1 to LPL).

### Data and PET-CT analysis

At fixed time intervals of 25 minutes duration during baseline and during cold exposure (after injection of [^18^F]FDG) energy expenditure was calculated. We use the term CIT instead of classical NST for the increase in energy expenditure (as a percentage) during cold exposure, as respiratory muscle contraction was involved. These time periods were also selected for analysis of cardiovascular and body temperature parameters. Two researchers (M.V. and G.V.) and a nuclear-medicine physician (B.B.) analyzed the PET-CT scan. In order to determine BAT activity, we measured the average [^18^F]FDG-uptake, known as the mean standard uptake value (SUV_mean_), and the total [^18^F]FDG-uptake (SUV_total_  =  SUV_mean_ multiplied by BAT volume) in BAT by manually drawing regions of interest. We considered fat tissue as BAT when the Hounsfield Units of the CT-scan were between -10 and 180. Moreover, a minimum of 1.5 SUV was used to classify the selected fat region as BAT. Furthermore, we analyzed average [^18^F]FDG-uptake (SUV_mean_) in multiple tissues in fixed volumes of interest according to the procedure described in Vosselman et al. 2013 [18]. The blood parameters presented in the subject characteristics were compared to normal reference values presented in the assay/kit information and with respect to the thyroid parameters, references values were obtained from the department of clinical chemistry at Maastricht University Medical Centre (Maastricht, the Netherlands). Since it was impossible to study the statistical difference between the two brothers, we compared the current results with data from previous studies in young adult men. To do so, we made boxplots of the data from young adult men and determined the interquartile range and the 95^th^ percentile. When the results of the twin were within the interquartile range observed in young adult men, we regarded both values (subject A and subject B) as comparable. When the score of one of the twin brothers was outside the interquartile range, this was regarded as different from each other. Furthermore, the score of the twins were considered as different from young adults when the score was outside of the 95^th^ percentile. This comparison has its limitations due to the differences in age between the young adult group and the twin brothers. Furthermore, it should be noted that the current cooling protocol lasted 30 minutes longer (2,5 hours versus 2 hours), and clothing was less (clo 0.1 versus 0.49). Therefore, temperature, skin perfusion, and cardiovascular data of the twin could not be compared with the young adults. However, for these measurements intra subject comparison was based on the measurement accuracy. BAT activity and CIT could be compared due to the use of the same individualized protocol in all subjects in which we cool the subjects to temperatures just above shivering, in order to obtain maximal NST and BAT activity in each subject (for more details on this protocol see [17]). The respiration values for skeletal muscle are represented as the average of two traces with standard deviation.

## Results

### Subject characteristics

Subject A had a lower fat percentage (13.7% versus 18%; normal range 11–22%), comparable fat free mass (69.4 kg versus 68.1 kg) and lower fasting triglyceride levels (subject A: 698 µmol/L versus subject B: 1060 µmol/L; normal range: 680–1880 µmol/L) than subject B (**Table 1**). Fasting glucose levels were equal (both 5.7 mmol/L; normal range: 3.61–6.11 mmol/L). Thyroid stimulating hormone levels were comparable, and within the normal range (A: 1.1 mU/L versus B: 0.9 mU/L; normal range 0.4–4.3 mU/L). Total T4 was slightly lower in subject A, and free T4 levels were comparable (total T4: A: 84 nmol/L versus B: 96 nmol/L; normal range: 60–150 nmol/L; free T4: A: 14.8 pmol/L versus B 14.7 pmol/L; normal range: 8–18 pmol/L). Furthermore, subject A had a more active lifestyle, although both were more active compared to the average found in young adults, indicated by the Baecke Questionnaire scores (total score: A: 11.4 versus B: 9.7; average young adults: ±8.2, derived from [19]). Interestingly, the monozygotic twin was genotyped with the polymorphism for uncoupling protein-1 (G/G). These G-allele carriers have been associated with an attenuation of UCP-1 mediated thermogenesis [20]. No polymorphism (no Arg64 allele) was present in the β3-adrenergic receptor.

**Table 1:** Subject characteristics.

	Subject A	Subject B
Height (cm)	183	184
Weight (kg)	82.2	87.0
BMI (kg/m^2^)	24.7	25.8
Fat percentage (%)	13.7	18
Lean mass (kg)	69.4	68.1
Fasting glucose (mmol/L)	5.7	5.7
Fasting triglycerides (µmol/L)	698	1060
Thyroid stimulating hormone (mU/L)	1.1	0.9
Total T4 (nmol/L)	14.8	14.7
Free T4 (pmol/L)	84	96
Resting Metabolic Rate (MJ/day)	8.1	7.8
Baecke Questionnaire Score (WI/SI/LI (total)*	3.1/4.3/4 (11.4)	2.4/3.5/3.8 (9.7)

*WI  =  work index, SI  =  sport index, LI  =  leisure time index,

### Physiological parameters and thermal sensation during cold exposure

**Figure 2:**
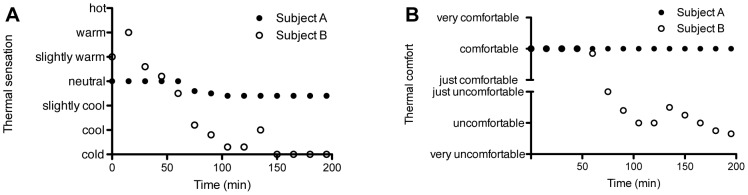
Visual Analog Scale (VAS) of thermal sensation and comfort during the mild cold experiment. This figure illustrates the thermal sensation (**A**) and comfort (**B**) of both subjects during the mild cold experiment.

Interestingly, during the “shivering experiment” on day one, both subjects did not reach the point of shivering. Normally, in such conditions with temperature drops from 31 to 13°C, young adults and elderly start shivering [21], [22]. The next day we maximally decreased ambient temperature and temperatures dropped to maximally 12°C. Again, no shivering was observed during this cold experiment. Mean skin temperature was comparable between the subjects during baseline (A: 33.22°C versus B: 33.40°C, **Table 2**), however during cold exposure mean skin temperature decreased more pronounced in subject B compared to subject A (A: 27.57°C versus B: 25.95°C). This decrease was predominantly seen in the proximal region (A: 29.36°C versus B: 27.81°C). Both subjects had comparable vasoconstriction in the hand (A: 91,7% versus B: 91,3%) and toe (A: 96% versus B: 96.4%), although subject A had a slightly lower distal temperature (A: 21.43°C versus B: 22.34°C). Subject A showed more vasoconstriction in the underarm (A: 63.3% versus B: 8.8%) and abdomen (A: 7% versus B: −8.8% (vasodilation)). Core temperature decreased in both subjects with a smaller decrease (−0.18°C) in subject A compared to subject B (−0.40°C). As the accuracy of the measurement is ±0.1°C and due to the small range within core temperature is held, we interpreted the difference of −0.22°C as physiologically different. There was a clear difference in cold sensation and comfort between both subjects. Both subjects experienced the thermal environment as neutral during baseline, however subject A reported neutral to slightly cool during the mild cold period, whereas subject B felt cold (**Figure 2**). Furthermore, subject A reported that he was comfortable with these temperatures during the entire experiment, whereas subject B felt between uncomfortable and very uncomfortable. Cold exposure slightly increased heart rate in subject A (baseline: 46 beats/min versus cold: 52 beats/min), whereas it slightly decreased in subject B (baseline: 51 beats/min versus cold: 47 beats/min). Both subjects were bradycardic during rest conditions. Mean arterial pressure increased to a similar extent upon cold exposure in both subjects (A: from 93 mm/Hg to 109 mm/Hg; B: from 99 mm/Hg to 111 mm/Hg). After 90 minutes in the cold, plasma free fatty acids increased during cold exposure in both subjects (A: baseline 625 µmol/L versus cold 771 µmol/L; B: baseline 264 µmol/L versus cold 705 µmol/L), whereas plasma glucose, insulin and epinephrine concentrations slightly decreased (**Table S1**). Plasma norepinephrine increased in both subjects, however, the concentration during cold was markedly higher in subject B (A: 554 ng/L versus B: 1016 ng/L), suggesting greater sympathetic activity.

**Table 2:** Metabolic, cardiovascular and thermoregulatory parameters during mild-cold experiment.

	Baseline		Cold	
Parameter	Subject A	Subject B	Subject A	Subject B
Energy expenditure (kJ/min)	5.51	5.47	7.71	7.76
Respiratory quotient (VCO2/VO2)	0.82	0.87	0.82	0.88
Heart rate (beats/min)	46	51	52	47
Systolic blood pressure (mmHg)	124.3	127.3	142.3	149.3
Diastolic blood pressure (mmHg)	78.0	84.3	93.0	93.0
Mean arterial pressure (mmHg)	93.4	98.7	109.4	111.0
Vasoconstriction hand (%)	0	0	92	91
Vasoconstriction toe (%)	0	0	96	96
Vasoconstriction arm (%)	0	0	63	9
Vasoconstriction abdomen (%)	0	0	39	−7
Core temperature (°C)	36.57	36.76	36.39	36.36
Mean skin temperature (°C)	33.22	33.4	27.57	25.95
Proximal temperature (°C)	33.51	33.37	29.36	27.81
Distal temperature (°C)	32.99	32.65	21.43	22.34
Gradient proximal, distal (°C)	0.51	0.72	7.93	5.47

### Cold-induced thermogenesis and [^18^F]FDG-uptake in BAT and skeletal muscle

**Figure 3:**
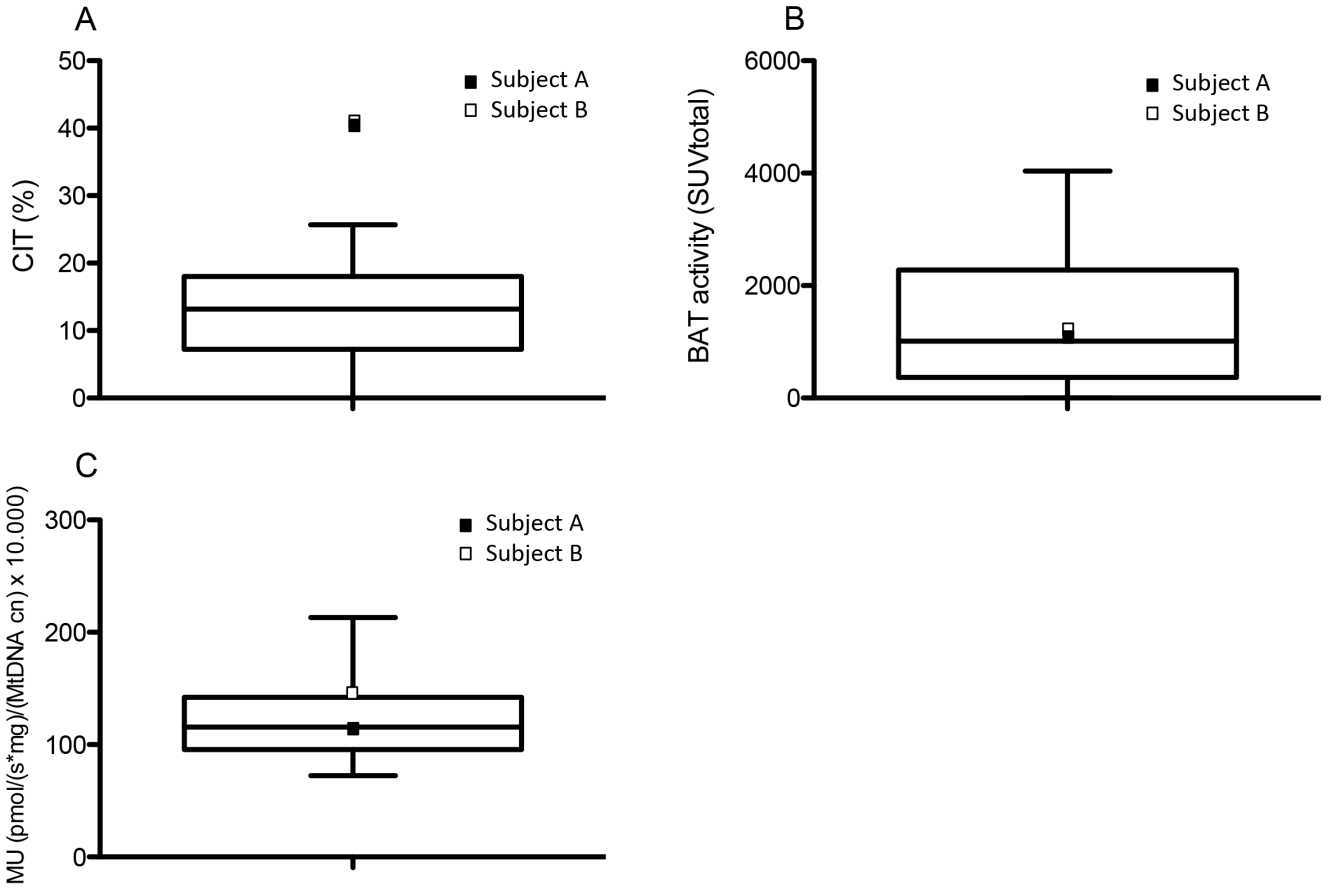
Comparison of CIT, BAT activity and skeletal muscle intrinsic mitochondrial uncoupling between the monozygotic twin and young adult men. Boxplots indicating the median and interquartile range of CIT (A) and BAT activity (B) found in young adult men during previous studies [6], [17], [18], [29] (CIT n = 43; BAT activity n = 45). C) Boxplot showing the skeletal muscle mitochondrial proton leak in young adult men (n = 30) from a previous study (n = 8) and unpublished results (n = 22). In each boxplot subject A is indicated as a black square and subject B as an open square. The whisker bars represent the 5^th^ (lower) and 95^th^ (upper) percentile.

**Figure 4:**
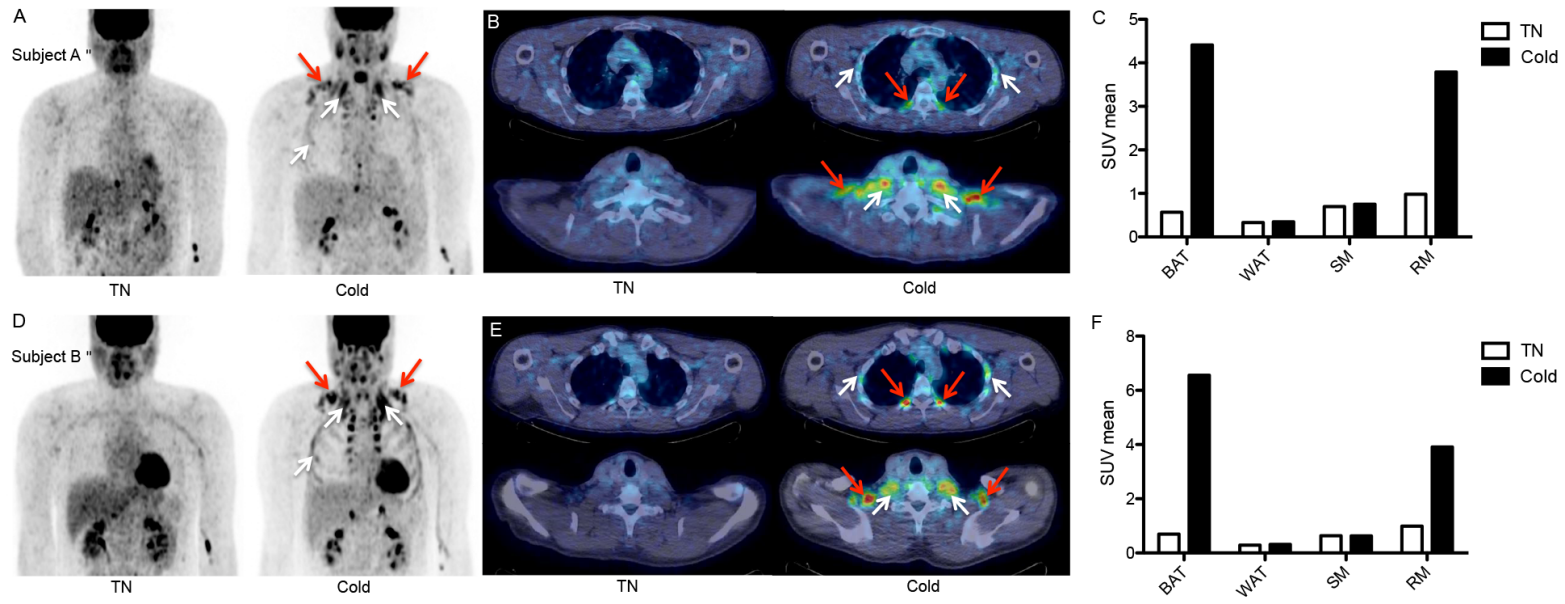
Brown adipose tissue and respiratory muscle activity during the thermoneutral and cold exposure experiment. A, D ) PET images during thermoneutral (left) and cold (right) conditions showing [^18^F]FDG-uptake e in brown adipose tissue (BAT; red arrows) and respiratory muscles (RM; white arrows). **B, E**) Transaxial slices of subject A (5 mm thick) of thoracic area (upper) and supraclavicular area (lower) demonstrating BAT activity (red arrows) and RM activity (white arrows). **C, F**) [^18^F]FDG-uptake (SUV_mean_) in BAT, white adipose tissue (WAT), skeletal muscle (SM), and respiratory muscles (RM) during thermoneutral and cold conditions.

Both subjects increased energy expenditure to a similar extent in the cold (A: from 5.51 kJ/min to 7.71 kJ/min versus B: from 5.47 kJ/min to 7.76 kJ/min, **Table 2**), resulting in a CIT of 40.1% and 41.9% for subject A and B, respectively. These values were clearly higher compared to the increase in energy expenditure we observed during mild cold experiments in young adult men (interquartile range: 7.2–18%; 95^th^ percentile: 25.7%, **Figure 3A**). However, it should be noted that the CIT in the current experiment is not equal to classical NST, as the twin used respiratory muscle isometric contraction to generate heat as well. As expected, both subjects A and B showed no active BAT during the thermoneutral experiment (**Figure 4A and 4D**). Interestingly, cold exposure led to a comparable increase in BAT activity, although it was slightly higher in subject B (A: 1144 SUV_total_; B: 1325 SUV_total_). These values are regarded as comparable since they both fall within the interquartile range (368−1930 SUV_total_; 95^th^ percentile: 4036 SUV_total_) of BAT activity found in young adult men (**Figure 3B**) [17], [18]. Thus, BAT activity is regarded as comparable to the values found in young adult men. Brown adipose tissue was present in the neck-, supraclavicular-, paravertebral-, and the perirenal area, which is comparable to the BAT distribution observed in young adults. We also determined [^18^F]FDG-uptake in WAT and skeletal muscle (SM) (**Figure 4C and 4F**). We did not observe any differences between the two subjects in glucose uptake in these tissues during both the thermoneutral (SM A: 0.7 SUV_mean_ versus B: 0.64 SUV_mean_; WAT A: 0.33 SUV_mean_ versus B: 0.22 SUV_mean_) and mild cold experiment (SM A: 0.75 SUV_mean_ versus B: 0.63 SUV_mean_; WAT A: 0.35 SUV_mean_ versus B: 0.24 SUV_mean_).

The cellular respiration data of the m. vastus lateralis fibers revealed that the state 4 respiration, reflecting mitochondrial proton leak per mitochondrion, was lower in subject A compared to subject B (A: 113.35±18.11 pmol/(s*mg)/(MtDNA copy numbers) x 10.000 versus B: 147.45±5.28 pmol/(s*mg)/(MtDNA copy numbers) x 10.000). The mitochondrial proton leak in the Iceman was within the interquartile range we observed in young adult men (n = 30) from a previous study and non-published data (interquartile range: 95.57–142.13 pmol/(s*mg)/(MtDNA copy numbers) x 10.000; **Figure 3C**) [6]. Subject B thus had a higher mitochondrial proton leak compared to his brother, as it was outside the interquartile range. However, it was not different from young adult males (95^th^ percentile: 213.13 pmol/(s*mg)/(MtDNA copy numbers) x 10.000).

Interestingly, both subjects immediately switched to the g-Tummo meditation/breathing technique at the onset of cold exposure, and although subject B does not practice g-Tummo meditation regularly, highly similar breathing patterns were observed (visual recognition). Therefore, we decided to analyze the [^18^F]FDG-uptake in two important respiratory muscles (RM), the mm. intercostales interior and exterior and the m. scalenus. Glucose uptake increased fourfold in these muscles during the cold experiment compared to thermoneutral conditions (**Figure 4C and 4F**), likely explaining a part of the high CIT levels. It is known that the energy cost of breathing is around 1–2% of total oxygen consumption, and that when ventilation increases, the oxygen consumption per liter of ventilation increases [23]. It might thus be that over 10% of the CIT is explained by increased cost of breathing.

## Discussion

The present study investigated the metabolic and insulative responses during mild cold in the so called ‘Iceman’, who has a lifestyle with frequent exposure to extreme cold, and in his monozygotic twin brother who is not frequently exposed to these extreme conditions. It was hypothesized that the Iceman would have a greater CIT and BAT activity. However, we found comparable CIT, BAT activity, and vasoconstriction in the extremities (hand and toe) during mild cold exposure and a slightly lower skeletal muscle mitochondrial proton leak in the Iceman compared to his twin brother.

Based on the frequent exposure of the Iceman to extreme cold, we expected to find greater BAT activity in the Iceman compared to his twin brother. It can be that these (short-term) extreme cold challenges do not recruit BAT. BAT is likely to be important during extended periods of mild cold exposure [24], and can maintain long periods of heat production. BAT thermogenesis has adaptive value because it is less exhaustive compared to shivering, due to its capacity for mitochondrial uncoupling via the specialized uncoupling protein 1 (UCP-1). We have recently shown that an extended period of mild cold exposure recruits BAT. In that study we exposed young adults to mild cold (15–16°C) conditions for ten consecutive days [6], and found that both BAT activity and NST increased. It is thus likely that long periods of mild cold exposure are more effective in increasing BAT activity and CIT than single bouts of extreme cold exposure. On the other hand, an explanation for the lack of difference in BAT activity in both subjects might be that the twin had a G/G polymorphism for UCP-1, which could negatively affect the plasticity of BAT [25]. This polymorphism therefore might preclude the recruiting effects of extreme cold exposure on BAT activity. Yet, the amount of BAT activity was comparable to young adult men and is likely high for their age, as BAT is known to decrease with ageing. A study on the effect of age on BAT found active BAT in only 10% of the subjects between 50 and 60 years old [9].

With respect to the insulative response, comparable vasoconstriction was observed in the hand and toe. However, a relatively large vasoconstriction response was found on the arm in the Iceman. This indicates a better insulation in the proximal region. Furthermore, the drop in core temperature was lower in the Iceman (−0.18 versus −0.4°C), indicating that the Iceman is better capable to maintain his body core temperature during mild cold conditions. The effects of the frequent exposure to extreme cold of the Iceman were well reflected by the thermal sensation and comfort. The Iceman felt slightly cool and comfortable during the entire mild cold protocol, whereas his brother experienced it as cold and uncomfortable. Thus, even though they had comparable metabolic reactions, there was a difference in subjective response to cold. Finally, plasma norepinephrine concentrations indicate that the mild cold induced a greater sympathetic stimulation in subject B. The sympathetic stimulation of BAT therefore might have been greater in subject B.

An interesting observation was the high heat production during cold (>40%) in both subjects. In our studies with young adults we normally observe NST levels (without g-Tummo) between −10 and 30% [6], [17], [18]. Given the fact that BAT activity and the intrinsic capacity for mitochondrial uncoupling in SM was comparable to young adults, other tissues and/or mechanisms must explain the high increase in CIT. Possible mechanisms that have been suggested are futile calcium cycling, protein turnover and substrate cycling [26]. However, in this special case a likely explanation for the high CIT levels is the g-Tummo like breathing technique (and possibly meditation in subject A) used by both subjects [12]. G-Tummo meditation consists of a somatic (isometric muscle contraction) and a meditative component (visualization of flames). The somatic component, which consists of deep abdominal “vase” breathing has been shown increased body core temperature, likely via increased heat production in both experienced g-Tummo meditators from Eastern Tibet and non-meditators (Western people) [12]. The meditative component exerted by the g-Tummo meditators was capable of sustaining temperature increases for longer periods. The monozygotic twin exerted this breathing technique as soon as the cooling protocols started. Metabolic activity of the respiratory muscles was clearly shown by increased [^18^F]FDG-uptake in the respiratory muscles during mild cold, which was absent during the thermoneutral experiments. It is therefore likely that a great part of the increased CIT can be explained by this breathing technique and could thus be a potential mechanism to fight cold challenges. We were not able to measure the effect of meditation on CIT and BAT activity. Whether the visualization component, as found by Kozhenikov et al. [12], is capable of increasing thermogenesis via BAT activity remains unclear.

During the cold experiments both subjects did not shiver, whereas normally young adults do. However, they were very close to shivering, because immediately after the experiment shivering occurred. This was likely due to the redistribution of cold blood leading to an after drop in core temperature (subject A: 36.39 to 36.10°C versus B 36.36 to 35.95°C). Our results indicate that the delay of shivering might be due to their increased respiratory muscle contraction (because of gTummo meditation practices). This parallels the fact that exercising in the cold does not induce shivering owing to increased heat production [27]. The exact role of the meditative component on temperature perception remains unknown. Another interesting observation was the bradycardia in both subjects indicating a possible alteration in cardiac autonomic control. Whether this is due to their meditation/breathing technique or a certain genetic factor (e.g. [28]) cannot be answered.

It should be noted that it is not possible to perform proper statistical analysis in a case study. The present results do provide new indications and ideas, which should lead to follow-up research. However, this case study is unique due to the identical genetic background of the subjects, which makes it possible to attribute the differences in results to lifestyle characteristics. In summary, we here show that the ‘Iceman’, who has a lifestyle with frequent exposure to extreme cold, does not have a greater heat production and BAT activity during mild cold conditions compared to his non-acclimatized monozygotic twin brother. Hence, the lifestyle of the Iceman does not affect the metabolic response to mild cold. The Iceman did have a relatively high insulative response and his thermal sensation and comfort were less affected by cold exposure. Based on these findings, it could be hypothesized that frequent but short term extreme cold exposure is less effective in BAT recruitment compared to longer periods of mild cold exposure. Interestingly, both brothers showed a high cold-induced heat production compared to previous mild cold studies in young adults. This was likely caused by contributions of both BAT activity and high levels of respiratory muscle contraction associated with g-Tummo meditation. G-tummo like breathing may thus be responsible for heat production in the cold in addition to classical NST.

## Supporting Information

Table S1 **Blood parameters during the mild cold experiment.** This table demonstrates several blood hormones and metabolites during baseline and at 90 and 150 minutes after the onset of cold exposure in both subjects.
(DOCX)
