# Supplementary material for: Frequent Extreme Cold Exposure and Brown Fat and Cold-Induced Thermogenesis: A Study in a Monozygotic Twin
Source: PLoS One. 2014 Jul 11;9(7):e101653. doi: 10.1371/journal.pone.0101653 (PMC4094425; doi:10.1371/journal.pone.0101653)
Supplement: Table S1 — Blood parameters during the mild cold experiment. This table demonstrates several blood hormones and metabolites during baseline and at 90 and 150 minutes after the onset of cold exposure in both subjects. (DOCX) [file pone.0101653.s001.docx]

**Table S1: Blood parameters during the mild cold experiment**

| Baseline | | Cold 90 minutes | | Cold 150 minutes | |  |
| --- | --- | --- | --- | --- | --- | --- |
| subject A | subject B | subject A | subject B | subject A | subject B |  |
| Free fatty acids (µmol/L) | 625 | 264 | 771 | 705 | 881 | 901 |
| Glycerol free (µmol/L) | 97 | 57 | 112 | 142 | 139 | 184 |
| Triglycerides (µmol/L) | 698 | 1060 | 766 | 1083 | 782 | 1106 |
| Glucose (mmol/L) | 5.8 | 5.3 | 5.7 | 5.0 | 5.6 | 4.9 |
| Insulin (µU/mL) | 10.1 | 11.0 | 8.2 | 10.1 | 8.1 | 6.0 |
| Norepinephrine (ng/L) | 389 | 284 | 439 | 751 | 554 | 1016 |
| Epinephrine (ng/L) | 32 | 21 | 21 | 10 | 16 | 7 |
| TSH (mU/L) | 1.1 | 0.9 | - | - | - | - |
| Free T4 (pmol/L) | 14.8 | 14.7 | - | - | - | - |
| T4 (nmol/L) | 84 | 96 | - | - | - | - |
